# Aesthetic value protection and tourism development of the world natural heritage sites: a literature review and implications for the world heritage karst sites

**DOI:** 10.1186/s40494-023-00872-0

**Published:** 2023-02-13

**Authors:** Shirong Zhang, Kangning Xiong, Guangyu Fei, Haipeng Zhang, Yongbi Chen

**Affiliations:** 1 School of Karst Science, Guizhou Normal University/State Engineering Technology Institute for Karst Desertification Control, Guiyang, 550001 Guizhou China; 2 School of Culture and Tourism, Guizhou Education University, Guiyang, 550015 Guizhou China

**Keywords:** World Natural Heritage sites, Aesthetic value protection, Tourism development, Literature review, Karst

## Abstract

Recently, research on outstanding universal value (OUV) protection and tourism development at World Natural Heritage sites (WNHSs) has attracted scholarly attention. The aesthetic value of natural landscapes is a powerful driving force for tourism development. Using this approach as an entry point to study aesthetic value protection and tourism development will help to relieve the contradiction between protection and development and promote the sustainable utilization of heritage sites. However, no comprehensive literature review has examined research on aesthetic value protection and tourism development at WNHSs. To fill this gap, we used a systematic literature review framework, and bibliometric analyses of 194 journal articles were collected from the Web of Science (WOS) and China National Knowledge Infrastructure (CNKI) databases. The results show the following: (1) the overall fluctuating upward trend in the number of publications indicates that the research in this field is gradually expanding, the regions studied in the literature are mainly focused on China, Italy and Australia, and the research institutions conducting the research are mainly Asian universities. (2) The landmark research achievements mainly focus on theoretical foundation, model construction, monitoring and evaluation, technical measures and other dimensions and the implications for World Heritage karst sites (WHKSs), where theoretical research is the main focus, and that research on monitoring and evaluation, technical measures and model construction is relatively limited. (3) On this basis, 7 key scientific and technological issues are summarized and provide insights into future research directions for the conservation of aesthetic values and tourism development at WHKSs, and future research should strengthen knowledge of natural heritage conservation based on aesthetic value identification and focus on the theoretical basis of the coordination between the conservation of aesthetic value and tourism development at WNHSs. Research should explore the science and technology and measures associated with the coordination between aesthetic value conservation and tourism development, and reveal the mechanisms and paths for coordination between WNHS conservation and tourism development in karst areas.

## Introduction

World Natural Heritage sites (WNHSs) are natural areas of outstanding scientific and aesthetic value that developed during the evolution of the Earth [1], and they are characterized by having outstanding universal value (OUV). World Natural Heritage sites (WNHS) conservation has become a global undertaking, and OUV is an important aspect of heritage conservation [2, 3]. At the same time, WNHSs preserve the most complete and original appearance of nature's charm and craftsmanship; they are intuitive, vivid and comprehensive natural knowledge textbooks for a country or region, and heritage site tourism provides tourists with a full range of contact with and appreciation for the beauty of nature's stunning landscapes. In addition, WNHSs help shape the country’s image and promote tourism destination branding [4, 5]. As a special tourist destination, a WNHS is unique in the tourism industry due to the outstanding scientific and aesthetic value of its natural resources [6, 7]. However, the conservation and development of the OUV of a number of WNHSs face severe challenges, for example, natural disasters or tourism activities that threaten the integrity and sustainability of the aesthetic value of natural landscapes [8–11]. Therefore, how to coordinate the relationship between OUV protection and tourism development at a WNHS is gradually becoming the focus of some scholars' attention [12, 13].

Aesthetic value (criterion vii) is an important part of assessing OUV [14]. The United Nations Educational, Scientific and Cultural Organization (UNESCO) defines the OUV of the aesthetic aspects of WNHSs as follows: “superlative natural phenomena or areas of exceptional natural beauty and aesthetic importance” [15]. By July 2021, when the 44th Heritage Conference concluded in Fuzhou, China, 213 WNHSs and 39 mixed sites (including Natural Heritage and Culture Heritage) around the world had been successfully added to the World Heritage List (WHL)*.* Among them were 146 heritage sites meeting the aesthetic value criterion [16], which shows that most WNHSs with aesthetic value are high-quality tourism landscape resources. Whether it is the popularity of mass leisure travel and sightseeing tourism, or the promotion of ecotourism and sustainable tourism, as international brands with world-class natural beauty and important aesthetic value, these sites usually become preferred tourism purpose regions for tourists [17]. This means that outstanding aesthetic value is an important driver of tourism and recreational visits to natural areas, which can be used as a vital starting point for building the brand of WNHSs, help give full play to the brand effect of heritage sites, broaden the choice of tourists to visit WNHSs as tourism purposes, and then promote the transformation of aesthetic value into economic value, providing an economic basis for the conservation and management of most WNHSs [18–20], especially in developing countries [21]. On the contrary, a decline in the aesthetic quality of natural landscapes can reduce human well-being [22, 23]. The importance of preserving the aesthetic value in the context of sustainable development was recognized by many countries at the Rio + 20 conference [24]. Incorporating the aesthetic value of natural landscapes into the goals and practices of environmental conservation not only maximizes the contribution of natural landscapes to human society but also strengthens support for the protection of WNHSs [25, 26]. Therefore, a literature review of aesthetic value conservation and tourism development can serve as a breakthrough in the study value protection and tourism development of WNHSs.

Facilitating the relationship between aesthetic value conservation and tourism development is an important prerequisite for promoting the conservation of natural landscapes and sustainable tourism at WNHSs. At the 34th Heritage Congress in Brazil in 2010, the World Heritage Committee (WHC) emphasized that tourism is both an opportunity and a challenge for World Heritage sites (WHSs) [27]. Tourism development at WNHSs is a double-edged sword that can cause damage to landscape aesthetic value and ecological threats while promoting economic growth. In the relationship between protection and tourism at WHSs [6, 28], aesthetic value protection and tourism development at WNHSs are both in opposition and agreement. Aesthetic value is the driving force of tourism development, and the protection of aesthetic value is the.

premise of tourism development. Tourism activities feed back into the protection of value, and the development of tourism plays a positive role in promoting the protection of aesthetic value. The aesthetic value of WNHSs is a manifestation of the earth’s processes, which are very attractive to visitors [29, 30], and developing tourism by utilizing the aesthetic value of the landscape can help, particularly in developing countries, improve financial revenues and lift local people out of poverty [31–34]. However, protecting aesthetic value from damage is an important prerequisite for the development of tourism activities. The natural beauty of a WNHS is irreproducible, and its landscape resources are extremely fragile. If not strictly protected, the natural beauty formed over the years will be destroyed, causing the landscape to lose its original charm and reducing its attractiveness to tourists [35–37].

In contrast, tourism development plays a positive role in the protection of aesthetic value, which is reflected in the fact that tourism income provides a financial guarantee for aesthetic value protection and encourages stakeholders to protect the area’s aesthetic value and the ecological environment [38, 39]. For managers, tourism income can be used for aesthetic value protection and improve their protection and management capabilities. Local community residents gradually realize the importance of maintaining a beautiful natural environment as, based on the benefits of tourism, their awareness of ecological protection improves [40]. For tourists, motivation mainly comes from their perception of the attributes and value of heritage tourism destinations [41]. When visitors have pleasant aesthetic experiences, they will self-regulate their behaviour to protect the natural beauty and ecological environment of a heritage site. However, there have been no literature reviews on the progress of research on the link and relationship between the conservation of aesthetic values and tourism development. Examining the progress of research on aesthetic value conservation and the relationship between aesthetic value conservation and tourism development is of great practical significance for enhancing ecological environmental protection and promoting sustainable tourism at WNHSs.

Karst landforms are widely distributed all over the world, covering 22 million km^2^, constitute about 25% of the land surface of the world [42–44]. By July 2021, 30 WHSs with karst characteristics had been inscribed on the WHL [45], and 22 karst WHSs met criterion vii [16]. These facts show that the unique topographic features of karst landforms and the comprehensive natural landscape formed by the ecological environment are rich in landscape types, unique landscape structures, unusual aesthetic features, and high levels of natural beauty, which often have outstanding aesthetic value [46]. Therefore, WHKSs have tourism value due to their aesthetic, scientific and other characteristics, and the regional benefits of tourism are obvious [43, 47]. However, the karst environment is fragile [48], and karst landscapes are highly sensitive to disturbances caused by human activities [49]. Irrational development and utilization, an excessive number of tourists, and a segmented tourism management approach can lead to environmental pollution, landscape resource destruction, and compromised visual effects. For example, karst groundwater is an important source of drinking water for more than 10% of the world's population [50], but tourism activities affect the impact of water tourism on aquatic ecosystems in a WNHS, including lakes, streams, shoals and waterfalls [51], and opening the cave to tourists also hinders the conservation of the natural heritage cave [52]. Therefore, hosting tourism activities at WHKSs is extremely controversial. In view of the contradiction between aesthetic value protection and the development of tourism at WHKSs, there is a lack of in-depth research on both. However, based on an examination of the literature, most studies have focused only on either the aesthetic value of WHKSs or how heritage sites are developed and utilized, and synergistic research on the two together is lacking. The progress of research on the conservation of aesthetic value and tourism development of WNHSs can provide inspiration for the development of scientific and reasonable aesthetic value protection and tourism development for WHKSs to realize the sustainable development of these sites.

Existing studies have mainly been reviewed from the perspective of the basic issues of WNHS aesthetic value description and application, the holistic protection and tourism development of WNHSs, tourism activities and stakeholders, and they have mostly analysed a single aspect of the literature. However, studying aesthetic value protection and tourism development at WNHSs is of vital practical significance for protecting WNHSs and supporting sustainable development. The organization of the present literature and knowledge system facilitates the self-reflective growth and reproduction of synergistic WNHS protection and tourism research [52]. However, a literature review on the study of aesthetic value conservation and tourism development at WNHSs is lacking. Therefore, to fill this gap, this article provides a systematic review and bibliometric analysis of the present findings from the literature on aesthetic value protection and tourism development at WNHSs. It reviews the main research progress (“Results” Section) and landmark achievements in aesthetic value protection and tourism development (“Main developments and landmark achievements” Section), including theoretical research, technical measures, monitoring and evaluation, and model construction, as well as the implications for the conservation and development oft WHKSs. It also proposes seven key scientific questions to be addressed (“Key scientific and technological issues to be solved and prospects” Section) and directions for future research based on existing studies to provide an objective and systematic review of the current state of research on WNHS conservation and tourism development.

## Data and methods

**Fig. 1:**
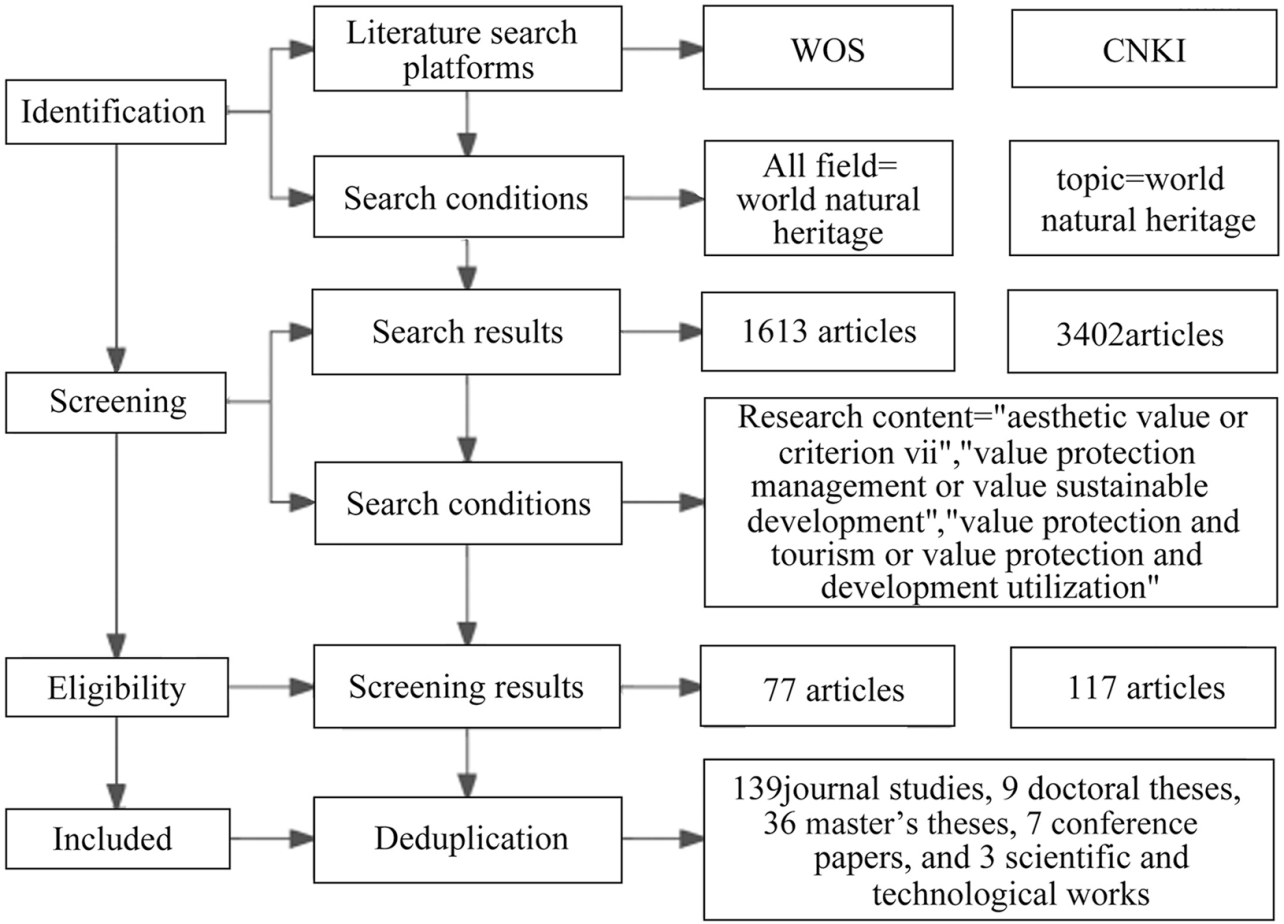
Process of the literature search and screening

The systematic review and bibliometric analysis of scientific information provided by databases has been applied to a variety of research fields, such as ecosystem services and ecological compensation of WHSs, heritage tourism, environmental protection and management [53–55]. Systematic reviews and bibliometric analyses, mainly through the analysis of previous rigorous studies, help scholars to familiarize themselves with the existing research results of the problem to be studied, to understand the progress and trends of related research, and to enable scholars to expand their research ideas and research methods and develop new research perspectives. The available articles and literature were obtained from the China National Knowledge Infrastructure (CNKI) database (https://www.cnki.net/) and Web of Science Core Collection (WOS) database (https://www.webofscience.com) and apply to the study of aesthetic value protection and tourism development at WNHSs. Our approach comprised four main steps (Fig. 1): identification, screening, qualification, and inclusion [56].

First, in the WOS, "World Natural Heritage" was used as the search term for the first search, and "aesthetic value or criterion vii ", "value protection management or value sustainable development", and "value protection and tourism or value protection and development utilization" were used as the search terms for the second search 77 articles were found. Second, in the CNKI database, "topic" was used as the search item, and "World Natural Heritage" was used as the search word for the first search. Using the results of this first search, "aesthetic value or criterion vii ", "value protection management or value sustainable development", and "value protection and tourism or value protection and development utilization" were used as the search words for the second search, and 117 articles were found. The search eliminated works outside of the maximum time range of the databases. Finally, the retrieved literature was screened based on the research content of this article, and the title, abstracts and body of the literature were analysed.

After deduplication, we a total of 194 studies related to aesthetic value conservation and tourism development at WNHS were obtained or were of reference significance. These studies included 139 journal studies, 9 doctoral theses, 36 master's theses, 6 conference papers, and 3 scientific and technological works. The top 10 contributors in number of articles of the topic were Kangning Xiong (17), Zhaoping Yang (12), Shizhen Xiao (11), Fang Han (8), Hui Shi (5), Zhaoguo Wang (5), Marshall Nadine (4), Keqin Sun (3), Yaohua Chen (3) and Xiaoqing Xu (3). When calculating the number of published articles by authors, all authors in the articles were accounted for regardless of the author’s order.

## Results

### Annual distribution of the literature

**Fig. 2:**
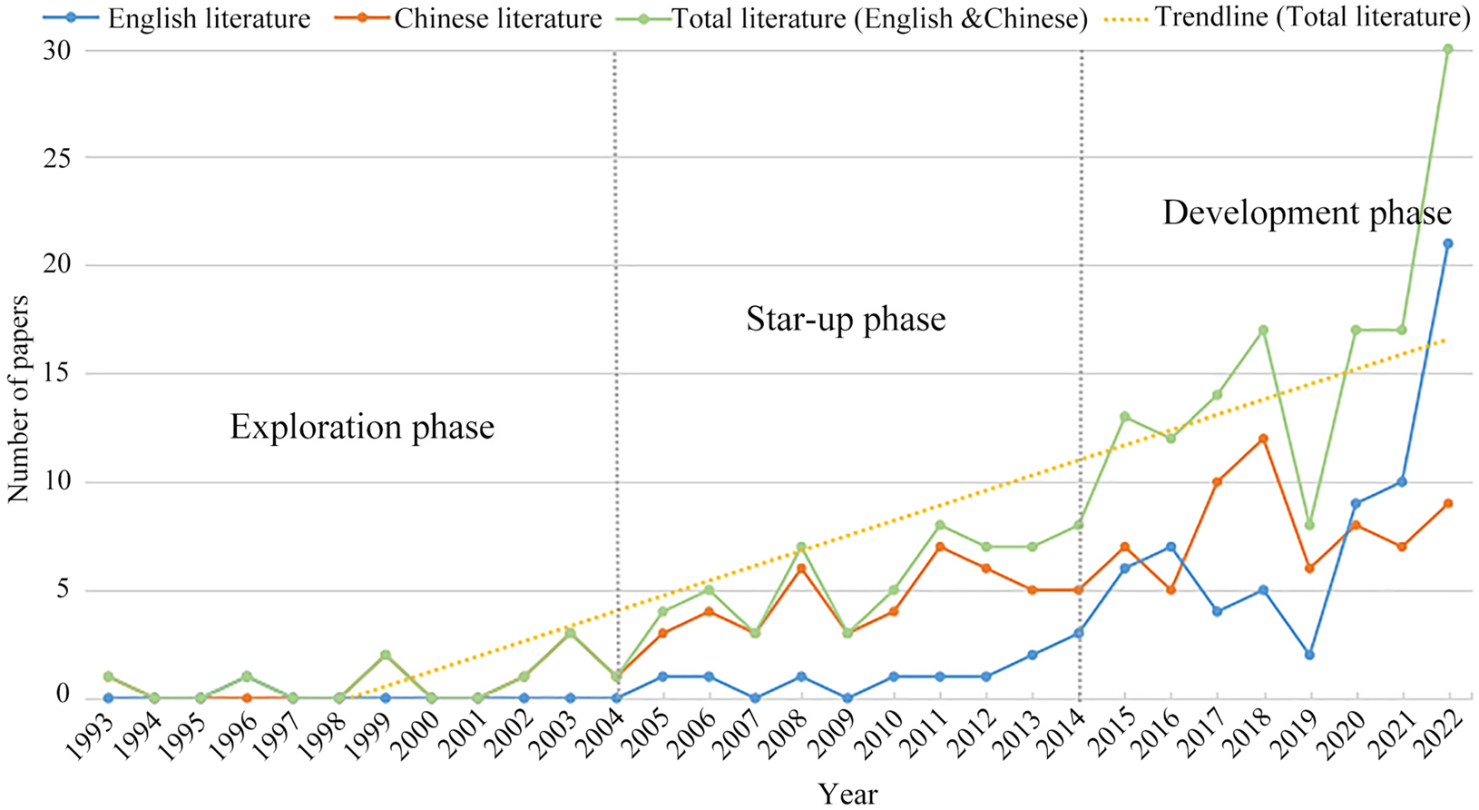
Trends in the paper related to aesthetic value conservation and tourism developing research of WNHSs by year until 31 December 2022

The annual distribution of studies on aesthetic value protection and tourism development at WNHSs, which shows a general upwards trend with a certain volatility, can be roughly divided into three stages (Fig. 2). The first stage was from 1993 to 2004, when the topic appeared sporadically in the literature, with the total number of studies not exceeding five per year, indicating that the stage is in the exploration phase. The second stage was from 2005 to 2014. The number of studies rapidly grew, and this stage was the starting period. The next stage showed a fast increase, which occurred in the 2015–2022 period. In general, the number of studies pertaining to aesthetic value protection and tourism development at WNHSs surged, reaching the highest level in 2022. Moreover, the trends in the Chinese literature and overall literature were basically the same (Table 1).

**Table 1:** Division of the research stages of aesthetic value protection and tourism development at WNHSs

Research stage	Background	Main characteristics
Exploration phase (before 2004)	The Convention Concerning the Protection of the World Cultural and Natural Heritage (hereinafter referred to as "the Convention"). The Convention was promulgated, which clearly stipulates that WHNSs must meet the value criteria and conservation management requirements. In the 1990s, the global economy recovered and tourism activities developed rapidly, and the tourist destinations were mainly heritage sites satisfying aesthetic value. However, with the threat of tourism activities to the natural beauty of the heritage site landscape, whether to carry out tourism has aroused controversy	The application of aesthetic value standard is controversial, and the exploration of the conservation of heritage as a whole are available, and there is no research literature on the conservation of aesthetic value. The study of tourism examples in WNHSs is relatively small and mainly focuses on theoretical research
Start-up phase (2005–2014)	At the beginning of the twenty-first century, there is a rapid development of domestic and foreign tourism, the world's pursuit of high-quality tourism that is accompanied by a serious environmental crisis. With the development of tourism, some scholars admit that the economic benefits generated by aesthetic value can be beneficial to the protection of WNHSs. However, the aesthetic value OUV of heritage sites is also threatened by the construction of tourism infrastructure and tourists. People gradually begin to discuss the protection of aesthetic value OUV from ecology and integrity of heritage sites	The depth of research has increased, with more conservation studies highlighting the universal values of WNHSs with actual cases. Conservation studies on aesthetic value are still lacking. The application of aesthetic value standards is greatly affected by subjective factors. Therefore, in 2005, IUCN rearranged the order of standards in the Operation Guide. Scholars began to make an in-depth interpretation of aesthetic value criteria, but the protection of aesthetic value is lack of pertinence and microscopical research. Researchers began to focus on the importance of sustainability theory in the synergy between conservation and tourism development of WNHSs
Development Phase (2015-present)	WNHSs have become a hot topic both at home and abroad, and the International Union for Conservation of Nature (IUCN) makes further revisions to the OUV criteria, reflecting a change in the concept of WHSs conservation, with heritage development and utilization based on the conservation of OUV. People beginning to value the question of how to improve coordination between conservation and tourism. The importance of the aesthetic value of heritage sites as an increase in human well-being and the importance of preserving aesthetic value were recognized	WNHS research is richer, the recognition of OUV is strengthened, the protection of other OUV including aesthetic value is beginning to be emphasized, and the concept of sustainable development is more emphasized in tourism at heritage sites. The synergistic development of aesthetic value conservation and tourism is in the exploration stage

### Content distribution of documents

Based on the literature classification method, this article classifies and summarizes the 194 papers reviewed as theoretical research, technical measures, monitoring and evaluation, model construction and other related studies (Table 2). Theoretical research accounts for approximately 49% of the total research volume, constituting the main part of the current research. It is followed by technical measures and monitoring and evaluation, accounting for approximately 17% and 16% of the total research, respectively. The number of other research types, such as model construction and experimental demonstrations, in the literature is relatively small, approximately 8% of the total articles. Based on the proportions of these studies, it is clear that the theory of aesthetic value protection and tourism development at WNHSs is becoming more mature, while technical measures, monitoring and evaluation, model construction and experimental demonstrations are in the continuous exploration and development stage.

**Table 2:** Type of literature content of aesthetic protection and tourism development at WNHSs

Type of literature content	No. of articles	Percentage (%)
theoretical research	96	49.48%
technical and measures	33	17.01%
monitoring and evaluation	31	15.98%
model construction	15	7.74%
Others	19	9.79%

**Fig. 3:**
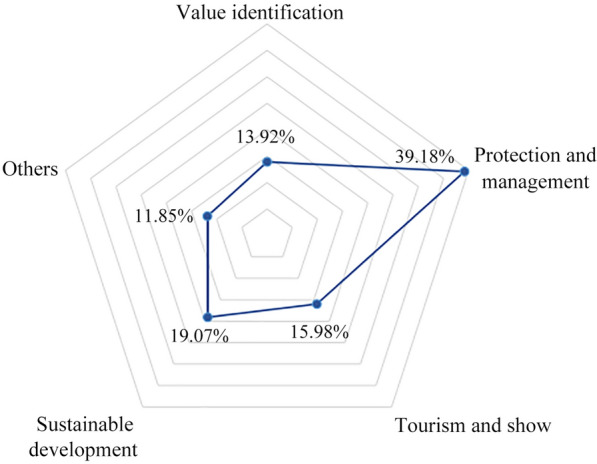
Literature by content

The Convention aims to identify, protect, preserve, and present WHSs with OUV and transfer them to future generations [57]. Therefore, in accordance with the original intent of the Convention, we divided the research content of the literature into value identification, protection management, tourism and present, sustainable development and other topics (Fig. 3). Research on conservation management and sustainable development accounts for approximately 60% of the studies in the sample, indicating that the protection and inheritance of heritage are popular research topics in the WNHSs field and that the most important attribute of heritage is the protection attribute. Tourism and present accounted for one-third of the research, indicating that in addition to the conservation attribute, in WNHSs research, people have gradually focused on the display of heritage for tourism purposes. However, value identification and other research on WNHSs accounted for a relatively small proportion of the literature.

### Country and institution distribution of the literature

We interpreted and analysed the distribution of the countries by using the occurrence frequency of each study area of related articles as the basis and then listed the main countries from high to low. China is the main research region, followed by Italy and Australia, with the South China Karst, Jiuzhaigou and the Great Barrier Reef (GBR) serving as hot research regions. Moreover, Poland, Switzerland, Vietnam and other countries are also involved in related studies. In general, the distribution of the study areas is positively correlated with the global distribution of WNHSs.

The institutional distribution of studies indicates that the highest number of publications is concentrated in Asian universities (Guizhou Normal University, Xinjiang Institute of Ecology and Geography Chinese Academy of Sciences, Chinese Academy of Sciences, Jishou University, etc.). Among the research institutions in China, most research occurs at universities in regions where WNHSs are located, and research in institutions outside of these areas is relatively rare. The distribution of scientific research on WNHSs in other countries is relatively fragmented. However, research is not limited to universities; research is also conducted by research institutes, local heritage management agencies and other research units related to the design of heritage conservation and management.

## Main developments and landmark achievements

Theoretical research is the basis for guiding the conservation of aesthetic values and tourism activities in WNHSs, and technical measures and monitoring evaluation are the guarantee and means for achieving coordination between the conservation of aesthetic values and tourism development. The construction of a model offers the opportunity to learn about and summarize the successful experience of conservation and tourism development in an existing WNHS and can provide a reference for the development of other OUV conservation and tourism synergies in heritage sites. Therefore, on the basis of the abovementioned research content definition, we conducted a systematic review of the landmark achievements of the relevant research on the relationship between aesthetic value protection and tourism development at WNHSs to improve the research systems in these areas.

### Theoretical research

*(1) Based on the lack of systematic evaluation criteria for the aesthetic value of WNHSs, qualitative and quantitative research on the evaluation of aesthetic value criteria was carried out, and a framework system for the evaluation of the aesthetic value of natural heritage was constructed, serving as a theoretical basis for coupling aesthetic value protection and tourism development at WNHSs.*

The identification and evaluation of the aesthetic value of WNHSs are the basis for achieving aesthetic value protection and tourism development. Because the aesthetic evaluation criterion (criterion vii) is subjective and lacks a systematic assessment method, there are great controversies and limitations regarding its application, which makes it inconvenient for the WHC to judge the value of heritage sites. The IUCN intends to standardize the empirical and subjective judgement of aesthetic OUV through an evaluation study of value criterion vii and to establish a complete and scientific evaluation system for the OUV of WHSs. First, to identify "superlative natural phenomena", with the support of the IUCN, Mitchell et al. conducted a deconstruction analysis of the OUV of natural beauty through the interpretative development of the criteria for the identification of aesthetic value since the enactment of the Convention. They published the work Study on the application of Criterion vii, IUCN World Heritage Study, in which the most "aesthetic" features are obtained by comparing properties of the same type or inscribed on the World Heritage List through the "analogical method" [58, 59], for example, Jon et al. and Xu et al. compared Mount Kumgang and the Pamir Plateau with other similar heritage sites on a global scale, and concluded that these two areas meet outstanding aesthetic value as well as other outstanding value, and have the potential to be declared WHL. It provides the scientific basis for the nomination of these two areas as WNHSs [60, 61]. Second, it is difficult to evaluate "exceptional natural beauty and aesthetic importance" in the aesthetic value criteria because they are influenced by subjective aesthetic psychology and other complex factors. Therefore, a universal aesthetic criteria evaluation has not yet been issued internationally. In 2019, Ha & Yang took Xinjiang Tianshan as an example and constructed an evaluation framework for "natural beauty and aesthetic importance" in the aesthetic value of natural heritage by taking the characteristics and attributes of the aesthetic value elements of natural heritage, the theory of IUCN aesthetic value assessment methods, and the geomorphic landscape and the biological landscape as aesthetic evaluation factors [30], making a qualitative leap in the application of value criterion vii and contributing to the quantitative assessment of the aesthetic value of WNHSs.

In addition, Zhao & Xu deepened Bi’s rational understanding of "natural beauty" and "aesthetic importance" in criterion vii by analysing the application practices of criterion vii in different regions of the world [62]. Xu et al. interpreted and applied the research on value criterion vii, enriching the research on the OUV of WNHSs [63]. Zhang et al. made a comprehensive review of the natural beauty and aesthetic value of world heritage, and combined with the characteristics of WHKSs, proposed effective research methods, which provided ideas for formulating an aesthetic value oriented heritage protection management system [64]. Based on the construction of the aesthetic value evaluation system, the characteristic elements and value carriers of the aesthetic value of WHKSs can be identified, and their aesthetic value can be quantitatively described to formulate strategies for the conservation of the aesthetic value of WHKSs landscapes and to provide a scientific basis for tourism activities.

*(2) In view of the lack of value carrier identification in the aesthetic evaluation and OUV conservation and management of WNHSs, a theoretical study on the protection and utilization framework based on aesthetic value identification is carried out to provide a theoretical reference for coordinating aesthetic value conservation and tourism development.*

In the Operational Guidelines, a tool for WHSs protection and management, namely, the "value-carrier-carrier characterization" pathway of WH value identification, is proposed, laying the foundation for the protection and management of OUV. In 2008, the IUCN published the Criteria for Outstanding Universal Value of World Heritage Sites, which standardized the elaboration of the OUV and its carrier identification [65]. In the same year, the World Commission on Protected Areas (WCPA) published Guidelines for the Management Planning of Natural World Heritage Sites, which emphasized that "the statement of OUV of the WHSs, the application of criteria, the statement of authenticity and integrity are closely related to the protection and management of key elements". This quote indicates that the conservation of WNHSs is based on identifying their OUV [66] and that conservation studies based on identifying aesthetic value are particularly important, as aesthetic value is one of the important criteria of OUV.

*(3) To solve the contradiction between protection and tourism at WNHSs, multidisciplinary and multifaceted research on heritage conservation and development and utilization has been carried out, and a theoretical system of sustainable development and coordinated development has been constructed, laying a theoretical foundation for coordinated research on aesthetic value protection and tourism development.*

Research on WNHSs is interdisciplinary, and to achieve value protection and tourism development of WNHSs, theories and disciplines such as coordination, sustainable development, geography, and tourism need to be integrated to promote the sustainable development and inheritance of heritage sites. The concept of "sustainability" first appeared in the Operation Guide in 1994 in the field of heritage [67]. In 2002, the Budapest Declaration meant that the theory of sustainable development was formally applied to the protection and development of heritage sites. In 2012, in the goals of the Action Plan of the World Heritage and Sustainable Tourism Project adopted by the WHC, even more emphasis was placed on supporting heritage sites in adopting meticulous and responsible sustainable tourism methods to protect the OUV of WHSs. In 2014, to better guide the healthy development of WHSs, the World Heritage Centre released the World Heritage Sustainable Tourism Toolkit [68]. Sustainability theory plays an indispensable role in promoting the protection and tourism development of WNHSs. WNHSs are common goods of all humankind, and the purpose of their protection and utilization should be to achieve an increase in public welfare, which in turn will promote the overall development of human society [69].

For reconciliation and to ultimately achieve coordination [70], cooperation theory holds great significance in promoting the protection and development of WNHSs. Chen argued that the management, protection and development of WHSs should follow the principle of being "based on protection, scientific planning, rational development and sustainable utilization" to make protection and development occur synergistically [71]. Chen et al. used mainstream and mature strategic management ideas to construct a strategic management theoretical framework for the coordinated protection and tourism development of WNHSs [72], and they applied the framework to an empirical study of Langshan. Lin used Chinese heritage sites as an example to construct a two-stage sustainable development model to empirically study the importance of a good balance between economic development and ecological protection for sustainable development, and the balance between conservation and sustainable development of heritage sites requires the necessary cooperation and strategies of relevant institutions, practitioners and communities [73]. The protection of the fragile environments of WHKSs needs to be based on multidisciplinary and multiangle knowledge integration, and the concept of sustainable and coordinated development needs to be integrated to better promote the protection of their aesthetic value and sustainable development.

### Technical measures

*(1) To solve the problems of aesthetic value protection and tourism exhibition of WNHSs, scientific technologies such as "digital protection management information platforms", "remote sensing, geography systems, and global positioning systems" (hereinafter referred to as 3S) visual field analysis technology, and "3D" virtual tourism are used.*

A WNHS is characterized by sustainability, vulnerability, nonrenewability, and inheritance. Additionally, it is vulnerable to natural and man-made impacts and has a fragile ecological environment. Therefore, it is necessary to adopt science and technology to promote the protection and tourism development of heritage sites. Hu et al. outlined a variety of digitally processed virtual technologies for protecting world natural and cultural heritage [74], such as image rendering and reconstruction, image retrieval and restoration, copyright protection and immersive virtual systems. From the perspective of protection and development, Huang applied the theory of coordination to empirically prove the principle of the synergistic development of "Digital Jiuzhai" and its effect, proposing measures guaranteed to strengthen the synergistic effect [75]. Xiao et al. analysed the impact of establishing a glass bridge as part of the Wulingyuan tourism project on the aesthetic value of the site based on geographical information system (GIS) technology [76]. Aldighieri et al. used the San Lucano Valley in Italy as an example to develop the Openalp 3D virtual technology platform using information space technology, which can learn about geomorphological value and appreciate the beauty of the natural landscape of the WNHS, show the restricted areas of the site, and strengthen the interaction and connection between tourists and the heritage site, not only protecting and displaying the ecological and aesthetic value of the site but also increasing the ways in which people can have interactive tourism experiences [77]. These technical resources contribute to promote aesthetic value of heritage through digital communication platforms, increasing stakeholder awareness, education, awareness [78] and recognition of aesthetic value, and promote the sustainable conservation of WNHSs by stakeholders. WHKSs have important aesthetic value and other OUV aspects, with important ornamental and scientific educational significance. Using modern advanced displays, experience, interaction and other science and technology tools, people can understand the value of WHKSs, observe the unique landscapes and protect the fragile ecological environment. The coupling between aesthetic value and tourism development at WHKSs provides an effective method to achieve these aims.

*(2) To solve the problem that occurs when the aesthetic value of WNHSs is threatened and destroyed by natural disasters or human tourism activities, conservation measures and restoration techniques are proposed for natural heritage landscapes.*

Aesthetic value is vulnerable to the damage and destruction caused by natural factors, such as geological disasters, and human factors, such as tourism activities. Facing damage and destruction, it is necessary to take certain measures and use technology to restore the ecological environment, and the goal of ecological restoration is to restore the integrity of the aesthetic value. For the natural landscape in a WNHS, when its aesthetic value is affected by the natural factors of secondary disasters such as water erosion, landslides and debris flows, engineering and technical measures involving vegetation restoration, slope reinforcement and river reconstruction can be taken. Zhao et al. took Jiuzhaigou, which was affected by a debris flow, as an example and proposed a comprehensive debris flow prevention and control system composed of stable structures, blocking structures and sedimentary structures, as well as several other environmental measures, including placing galvanized wire cages in front of trees to reduce the impact of debris flows, building retaining walls and filling them with nutritious soil, planting shrubs and grass or planting green vegetation to beautify the landscape and increase the visual effect [79].

In addition to excessive grazing by heritage site residents, the construction of project facilities affects the visual beauty of the landscape, and damage to the natural landscape is caused by human activities and behaviours, such as picking and trampling of vegetation and littering by tourists. Measures include vegetation restoration technology, zoning protection, tourism scale control, and the reduction or demolition of nonessential facilities [80–82]. Tourists trample on the soil and vegetation in the site, walk in the water landscape, swim, and discharge wastewater from hotels, restaurants and toilets into lakes without treatment, and garbage is not properly treated. Surface trails were replaced with 60 km of elevated wood boardwalks, limiting visitor capacity, banning private cars and other ecological restrictions and restoration measures, and aquatic ecosystem protection achieved by integrating catchment management [83]. It effectively controls the water pollution of tourism to the environment and guarantees the aesthetic value of the water landscape in the heritage site.The fragility of karst WHSs makes their aesthetic value vulnerable to interference from and destruction caused by natural and human factors. Therefore, managers of karst WHSs should pay attention to the protection of the ecological environment and strictly restrict and prohibit the construction of tourism projects and facilities that will affect their aesthetic value. When the aesthetic value is affected, preventive measures and ecological restoration technology should be used in a timely manner.

### Monitoring and evaluation

*(1) Based on the requirements of aesthetic value protection and management at WNHSs, dynamic monitoring and evaluation research on aesthetic value conservation and management is carried out through the "non-expert method", providing an effective way to couple aesthetic value protection and tourism development.*

Monitoring and evaluation based on aesthetic value can provide a scientific basis for the value protection and tourism development of WNHSs. Marshall et al. monitored the aesthetic value of the GBR using ratings from non-expert stakeholders [84]. McIntosh et al. monitored aesthetic value by regularly collecting data and recording trends over time using reliable and consistent measures [85]. Petina et al. provided reef managers with information from monitoring the aesthetic value of the GBR through a nonexpert assessment approach and helped them to evaluate the status and trends of the aesthetic value of reef habitats to gain an understanding and take protective measures [86]. In addition, Le et al. and Scott et al. argued that the development of artificial intelligence technology has great potential for the long-term monitoring of the aesthetic properties of the GBR and for processing images representing large spatial scales [87, 88]. The evaluation method of monitoring the aesthetic value of the GBR can broaden the methods used to monitor the aesthetic value of WHKSs, and a comprehensive monitoring method is more conducive to protecting and managing aesthetic value and promoting sustainable tourism development of WHKSs.

*(2) Based on the requirements of value protection and tourism development at WNHSs, through the comprehensive utilization of "3S", a framework system for the monitoring, protection and demonstration of natural landscape heritage sites has been constructed, which provides a scientific basis for aesthetic value protection, management and tourism development.*

Monitoring and evaluation are means of effectively protecting and managing the value of WNHSs. Li constructed a monitoring and evaluation model and index system for WNHSs by investigating and studying the influencing factors of the OUV of Danxia Chishui, selecting indicators that can reflect the aesthetic value and geomorphic value of heritage sites or their carriers for field monitoring and evaluation [89]. Xiao et al. constructed a comprehensive monitoring system for WNHSs based on the example of Danxia Chishui, in which landscape changes and the aesthetic heritage landscape inventory were monitored based on surface remote sensing (RS) and fixed points, effectively monitoring and protecting the aesthetic value [90]. Shi et al. extracted and analysed the characteristic elements and interfering elements of landscape aesthetic value by using GIS and RS, constructed a heritage value conservation index system, and then proposed targeted measures for value conservation, providing a reference for the conservation monitoring of similar heritage sites [2].

Additionally, the method also facilitates the selection of tourism viewing at WNHSs, the conservation of heritage sites by grading and zoning, and a reduction in the negative impact of tourism activities on the ecological environment of WNHSs. For those sites that are difficult to access due to COVID-19 and complex landscapes, the role of remote sensing in heritage monitoring is important and irreplaceable, especially in karst areas with difficult terrain and complex geomorphology and magnificent natural landscapes [78], especially in karst areas with complex difficult terrain and magnificent natural landscapes, and the integrated monitoring system composed of "3S" technology cannot be ignored. It is necessary to use GIS to monitor the landscape aesthetic value of WHKSs, as GIS is an important tool for the effective management of value conservation and scientific tourism development.

### Model construction

*(1) To realize protection and tourism development, the WNHS ecotourism and sustainable model is constructed, which provides a model reference for aesthetic value protection and tourism development.*

Due to the fragility and nonrenewability of WNHSs, a suitable approach that balances protection and utilization must be found, and ecotourism is an optional method for achieving the coordinated development of the two. “Ecotourism” was first proposed by Ceballos, who stated that ecotourism is “environmentally responsible travel and visitation to relatively undisturbed natural areas, in order to enjoy and appreciate nature that promotes conservation, has low visitor impact, and provides for beneficially active socioeconomic involvement of local populations" [91]. The ecotourism model not only conforms to the requirements of the World Heritage Protection Convention but also meets the development trend of the world’s ecological civilization construction and tourism market. The concept of ecotourism can guide the development and utilization of WNHS landscape resources, aesthetic value, ecological environment protection and other activities at different scales. For example, the development of ecotourism can be used to educate the public, raise people’s awareness of the beauty and protection status of the natural landscape, and create considerable economic benefits. A karst landscape is an important ecotourism resource, and its unique natural beauty gives it high tourism development value [92, 93]. Sustainable development model is the inevitable choice to solve the problem between heritage protection and conflict [94]. Xiong et al. summarized the mode of conservation and sustainable development of the Libo WNHS, according to the principle of on the basic principle of "priority in protection, scientific development, sustainable use”, in accordance with the concept of heritage protection “core zone for protection, buffer zone for integration of management and development, upper catchment zone for combination of prevention and management”, and adopting protective measures of construction, development and infrastructure construction, and the Libo model conservation and sustainable development of heritage site was constructed [95]. As a method for effectively resolving the contradiction between protection and development, ecotourism has important reference significance for aesthetic value protection and tourism development at WHKSs.

*(2) To protect the aesthetic value and ecological environment of the WNHS landscape, the contradiction between heritage protection and tourism development should be resolved, and a recreational utilization mode of the natural heritage resources of protective development should be explored and created.*

Jiuzhaigou is famous for its colourful alpine lakes and spectacular calcified waterfalls, which attract many tourists every year. After negative impacts on the ecological environment and landscape occurred, the administration adopted a series of measures in a timely manner, such as returning farmland to forests and grassland and prohibiting indiscriminate mining and excavation, which steadily improved forest coverage and vegetation coverage, and a mottled colourful forest landscape became an important tourism resource of Jiuzhaigou. At the same time, it has implemented the protection and management mode of "travelling in the ditch and living outside the ditch" to solve the problems that threaten the sustainability of the landscape and ecological environment in the scenic spot, such as the discharge of domestic sewage and waste in local community tourism services [95, 96].

Furthermore, the construction of tourism infrastructure has been repeatedly discussed and demonstrated to meet the needs of tourism development while protecting the landscape and ecology. For example, the construction of roads considers the original style and features of the scenic spot and ensures that minimum damage to the mountains occurs based on the premise of meeting the basic requirements of construction. In addition, to divert people and vehicles, enhance the tourism experience and solve the problem of ensuring the tourism environmental capacity of the scenic spot is met, the Jiuzhaigou administration built pedestrian plank roads based on locally sourced stone masonry in the early stage and successively replaced, widened and upgraded the original plank roads in the later stage of development. These wooden plank roads stipulate tourist tour routes, reduce vegetation damage and soil erosion caused by human trampling, and protect the landscape and ecological environment of the heritage site while maximizing the tourist experience. The comprehensive management approach to the conservation-oriented development of Jiuzhaigou resolves the contradiction between tourism development and landscape resources, landscape aesthetic value and ecological environment protection. When carrying out tourism activities in WHKSs, it is necessary to adhere to a development model that prioritizes the protection of the landscape aesthetic value and ecological environment, rationally plans areas, and resolves the contradictions between residents, tourists and the tourism development of WNHSs to achieve coordinated protection and development.

*(3) To protect the aesthetic value and integrity of WNHSs and to coordinate the relationship between aesthetic value protection and tourism development, most natural landscape heritage sites have adopted a zoning development model.*

Zoning development is a widely used method in the protection, development and utilization of WNHSs, and zoning modes are different based on different regional cultural backgrounds and use functions. In 1973, under the guidance of concentric circle theory, landscape designer Foster divided Yellowstone Park into a core reserve, tourist buffer zone and dense tourist zone. Yan and Xiao mainly divided heritage sites into core protection areas, core environment areas, buffer areas and marginal areas, and they carried out protection, management, development and utilization at different levels and in different ways in different areas, which were mainly natural landscape heritage sites with large surfaces [97]. The aesthetic value and other values of a heritage protection area should be protected as a whole to maintain the original natural state of the ecosystem and natural landscape. On the premise of not destroying aesthetic value and other values, appropriate tourism activities can be carried out in a heritage exhibition area, but the scale of tourism facilities and the number of tourists should be strictly controlled to maintain the nature and coordination of the landscape.

The buffer zone, as an effective area for the protection of the OUV of the WNHSs and the coordination of site tourism, occupies a crucial position in the study of zoning models. Zhang et al. reviewed and studied the protection of WNHSs and the development of buffer zones, sorted out the relationship between buffer zones and the role of WNHSs in heritage tourism, and summarized the enlightenment of the synergy model of WHKSs protection and tourism [47], which provided ideas for constructing a zoning model of aesthetic value protection and tourism development. Zoning development is not only a method of effectively protecting aesthetic value and its carrier but is also a tourism development method for coordinating scenic spot resources and presenting aesthetic value. Due to the sensitivity of its background conditions and regional particularity, the managers of WHKSs should learn from the zoning development model and organically integrate its landscape resources with extremely high aesthetic value to achieve coordination and harmony between aesthetic value protection and tourism.

## Key scientific and technological issues to be solved and prospects

*(1) In view of the difficulty of constructing universal quantitative evaluation methods for aesthetic value, considering the differences in a subject's aesthetic perception and drawing on other methods for evaluating the aesthetic value of natural areas, a universal system for evaluating the aesthetic value of WNHSs is constructed.*

Due to the sensitivity of its background conditions and regional particularity, the management of WHKSs should learn from the zoning development model and organically integrate the site’s landscape resources with extremely high aesthetic value to achieve coordination and harmony between aesthetic value protection and tourism. Since people's understanding of beauty is influenced by many factors, the evaluation of the aesthetic value of WNHSs is subjective and mostly composed of unique landscapes, ecological environments and other complex factors that constitute their unique natural beauty. Therefore, it is difficult to unify the methods of understanding aesthetic value. At present, there are several methods for evaluating the aesthetic value of natural areas in other fields, including quantitative and qualitative methods [18, 98, 99], but most studies focus on vision and rarely consider the response of other senses to aesthetic evaluation. The aesthetic response of human beings to the environment is an overall sensory experience [100, 101], including the sense of pleasure aroused by relevant experiences such as smell, touch and sound [102]. Therefore, when examining the aesthetic value of a WNHS, different subjects and their different senses should be considered in the perception of aesthetic value, and a universal aesthetic value evaluation method should be used. At the same time, we can learn from other methods to evaluate the aesthetic value of natural areas, such as British landscape feature assessment (LCA) and American landscape resource assessment (VRM). The recognition and evaluation of the aesthetic value of WHKSs currently consist of qualitative descriptions in documents and application texts, and various quantitative research methods are lacking. In future research, if a universal aesthetic value evaluation is constructed by taking WHKSs as an example, it will have important reference value for the protection and tourism development of WNHSs.

*(2) Based on the ambiguous definition of the aesthetic value protection of WNHSs, a clear and universal definition of aesthetic value protection should be proposed based on empirical case research.*

At present, scholars have conducted relevant research on the integrity, authenticity and influencing factors of WHSs [103–105]. However, the definition of aesthetic value protection and how to carry out such protection in depth have not yet been clarified. IUCN considers the landscape visual factor to be an important component of natural value [106], and the conservation of natural landscape resources as a carrier of aesthetic value is one of the elements of aesthetic value protection. Aesthetic value is articulated in the Convention in terms of integrity as follows: the properties proposed under criterion vii should be of OUV and include areas that are essential for maintaining the beauty of the property [107], which emphasizes the integrity of the landscape to protect aesthetic value [108, 109].

Aesthetic value is often inseparable from ecological value [110], and some scholars believe that ecosystem processes and the characteristics of animals and plants play an important role in the composition of aesthetic value [111]. Furthermore, they believe that aesthetic value protection and development strategies should be formulated based on the protection of the ecological environment [2]. In addition, the evaluation criteria of aesthetic value need to be considered from the aspects of geological and geomorphological value, the characteristics of landscape combinations, and unique, rare, typical and other attributes of the landscape [58, 112–114]. Thus, the overall characteristics of the environment should be integrated to achieve the protection of aesthetic value.Therefore, in follow-up research, it is necessary to comprehensively consider the characteristics of the carriers of WNHSs, the characteristic elements of aesthetic value should be extracted and analysed, and the content of aesthetic value protection should be clarified to provide a scientific basis for the monitoring of WNHSs and the formulation of future conservation and management measures for managerial decision-makers. The content regarding the aesthetic value protection of WHKSs presents more complex characteristics. Exploring the definition of aesthetic value protection through the empirical research process has a certain reference value for the aesthetic value protection of other types of WNHSs.

*(3) To address the problems of the unclear identification of the aesthetic value elements of WNHSs, unclear protection goals and insufficient tourism presentation, empirical analysis is conducted with cases to identify and extract representative aesthetic value elements and alternative elements of WNHSs.*

A systematic and comprehensive identification system for aesthetic value has not yet been created, leading to an unclear identification of the aesthetic value carrier of WNHSs, unclear protection objects and goals, a failure to better present the landscape of WNHSs and show their OUV, and a failure to bring into play the enormous tourism potential brought by the aesthetic value of WNHSs, while value-based conservation has gradually been launched at the international level [115–117]. Focusing on case studies, adopting scientific and objective methods to identify the aesthetic value elements and clarify the value protection goals and value carriers can provide an effective way to protect and manage WNHSs, and the extraction of elements is an important prerequisite for the scientific development of tourism. The identification and extraction of representative aesthetic value elements and alternative elements of WNHSs can lay a foundation for the coordination of aesthetic value protection and tourism development. In future research on resolving the contradiction between the vulnerability and tourism development of WHKSs, using the identification and extraction of representative aesthetic value elements and alternative elements as a breakthrough can provide ideas for protection and tourism development.

*(4) In response to the problems posed by the generalized methods for the aesthetic value protection of WNHSs, the blurred boundaries between conservation and tourism, and the difficulty involved in the coordination of protection and tourism, combined with multidisciplinary knowledge, this paper systematically constructs a value protection system based on the identification of aesthetic value elements and proposes coordination strategies.*

Most WNHSs that meet the aesthetic value criteria have protection management attributes of scenic spots, national parks, nature reserves, etc., which are too general for enabling the protection of aesthetic value. It is particularly important to propose a system and measures for the protection of aesthetic value based on the identification of value elements for the development of this value. At the same time, clarifying the spatial scope and boundary of aesthetic value protection and tourism is an important prerequisite for the coordinated development of the two. In future research, it is necessary to learn from multidisciplinary theoretical knowledge, propose a collaborative strategy consistent with the aesthetic value protection and tourism development of WNHSs, and enrich the theoretical system of research. For a long time, the literature and materials on the aesthetic value of karst landscapes in China and elsewhere have shown a scattered trend. Research on the aesthetic value of WHKSs mostly stays at the level of the language and photographic descriptions in the "applicant text", and studies on the aesthetic value of WHKSs are lacking. In the research on conservation and tourism development, targeted value protection is the basis of tourism development. Tourism development can promote the protection of aesthetic value, and the coordinated development of the two is an important way to achieve the sustainable development of karst WHSs.

*(5)* *In view of the problem that the monitoring technology of the aesthetic value of WNHSs mostly stays at the level of qualitative research and analysis and does not propose a perfect value monitoring system, quantitative monitoring indicators and monitoring technology systems are built for the aesthetic value OUV of heritage sites, and scientific quantitative research methods and monitoring and evaluation models are formed.*

Space technology offers effective tools for monitoring the status of WNHSs [118]. Based on the research trend regarding protection management monitoring, the application of satellite RS, aerial RS, ground survey monitoring, along with big data, cloud computation, artificial intelligence, 5G communications and other heritage monitoring techniques has gradually increased, and some scholars have begun to focus their research on the monitoring of aesthetic value [119, 120]. However, most studies use qualitative or quantitative methods, and no quantitative research methods or monitoring and evaluation models have been formed. Moreover, monitoring data lack timeliness and are not shared, and an effective prediction of future trends is lacking. A WNHS that meets the aesthetic value standard attracts tourists as a high-quality tourist landscape resource. Effectively monitoring its value and carrier and building a systematic and perfect monitoring system have great significance, and doing so is conducive to promoting an improvement in the monitoring technology system of OUV. In addition, attention should also be paid to the regular reporting, conservation status and reactivity monitoring projects implemented by the WHC [118], to track the conservation status of the aesthetic value of the WNHSs and form a systematic aesthetic value monitoring system. The construction of a systematic monitoring and evaluation system is conducive to better protecting and managing WHKSs and can provide scientific support for follow-up tourism activities.

*(6) To address the lack of research on different stakeholders’ perceptions of the coordination between the aesthetic value of WNHSs and tourism development, taking tourists as an example to build a theoretical model of stakeholders' perceptions of this coordination, we discuss the mechanism of the influence of tourism on the aesthetic value protection of WNHSs.*

There are multiple stakeholders involved in the process of aesthetic value protection and tourism development at WNHSs. Each stakeholder has different resources and powers, as well as different motivations, goals and ways to participate in heritage value protection and tourism development. In the process of coordinated development, they are prone to encounter conflicts of interest. Coordinating the interests of stakeholders in protection and tourism development and unifying stakeholders in the harmonious development of heritage sites have become urgent issues for most WNHSs. Currently, most studies conducted on the conservation of WNHSs include communities as the most important stakeholders [121–123]. Nian et al. established a tourist perception system of OUV based on the relationship between community attachment, value perception, and service quality [124]. However, few studies have focused on the coordinated perception of value and tourism development from the perspective of managers, developers, tourists and other stakeholders. The environmentally responsible behaviour of tourists, who are one of the main stakeholders in tourism, is crucial for reducing the negative impacts on the sites visited [125]. Future research needs to pay more attention to the factors that influence the impact of tourist behaviour on the aesthetic value of heritage and the impact mechanisms. Due to the complexity of the karst environment, researchers and decision-makers encounter various difficulties in obtaining public perceptions of and preferences for karst landscapes [48]. Therefore, it is necessary to build a theoretical model of the coordinated perception of different stakeholders regarding aesthetic value protection and tourism development.

*(7) In view of the problems posed by the fact that aesthetic value protection and tourism development at WNHSs are difficult to coordinate and there is a lack of coordination model research, case studies and technical measures that meet the aesthetic value standards should be strengthened, the “win–win” of the two should be realized, and an exemplary coordination model of protection and development should be established.*

As one of the OUV criteria satisfying the WNHSs, aesthetic value makes a profound contribution to tourism value. However, there is a long way to go to realize the coordinated development of aesthetic value protection and tourism. Regarding the progress in current research, research is mainly based on the theory and synergistic model of the overall protection and tourism of WNHSs from a macro perspective [126], and microlevel research on the coordinated development of specific OUV protection and tourism is lacking. Meanwhile, empirical case studies of technical measures and coordination models have been conducted. In 2014, the IUCN proposed the OUV of WHSs and formed a sustainable protection management technology system based on modern science and technology. With more mature technical measures, there will be an important breakthrough in the coordination of aesthetic value protection and tourism. Establishing an exemplary collaborative development model will also be more conducive to the protection and sustainable development of WNHSs. Prominent karst landscapes are important for attracting tourists for regional development; they can create income for the local economy, provide employment opportunities for residents, and achieve the goal of sustainable heritage site development. In addition, there is an intricate relationship between value protection and tourism at WHKSs, and the corresponding coordinated development of aesthetic value protection and tourism is proposed. The technical measures and models discussed in this study can serve as a model for the sustainable development of otherWNHSs.

## Conclusion and future research

In this study, we implemented a comprehensive literature review by analysing 158 articles retrieved from Web of Science and CNKI. The main conclusions are as follows: (1) research on aesthetic value conservation and tourism development at WNHSs is generally on the rise, the regions studied in the literature are mainly focused on China, Italy and Australia, and the research institutions are mainly Asian universities. The research hotspots include the South China Karst, the Tian Shan of Xinjiang and the GBR of Australia. (2) Based on these studies, the main landmark achievements can be divided into five main aspects, including theoretical research, technical measures, monitoring and evaluation, model construction and others of the WNHSs, and the content is mainly focused on protection and management. (3) After further study, this paper proposed seven key scientific and technology issues that remain to be solved from this literature review, and these seven issues are hot research topics worthy of future in-depth exploration.

This study has summarized seven key scientific and technological issues of the aesthetic value protection and tourism development of WNHSs and the implications for WNHKSs, and the future directions for research on aesthetic value protection and tourism development at WNHSs can be considered from the following perspectives: how to quantify aesthetic value and build a set of comprehensive and universal aesthetic value evaluation models, how to build a protection system based on aesthetic value identification, how to alleviate the contradiction between aesthetic value protection and tourism development faced by WNHSs, how to achieve trade-offs and coordinate value protection and tourism development, the formulate targeted technical measures and thorough conversation management systems to realize sustainable protection and show aesthetic value, and how to build a monitoring technology and integrated information platform for OUV at WNHSs. These issues require further multidisciplinary and multilevel comprehensive research. In addition, future research may combine qualitative and quantitative research methods, constructing a high-quality theoretical system and increasing empirical research, whether from a micro or macro perspective. Future research should seek new breakthrough points based on available research on protection and tourism to achieve sustainable development at WNHSs.

WHKSs nurture unique natural beauty and have great tourist value. However, the fragile ecological environment and the prominent contradiction between local economic development and heritage protection make the sustainable development of WHKSs face severe challenges, particularly in developing countries. Future research should focus on the coordination mechanism of transforming the aesthetic value into economic value, deepen stakeholders' understanding and recognition of the outstanding aesthetic value, explore technical measures to adapt to the synergy between the protection of aesthetic value and tourism development of WHKSs, establish an aesthetic value protection system of "value-carrier-protection" and monitoring information platform of WHKSs, build a sustainable development model with distinctive karst characteristics, and carry out pilot demonstrations.

## Data Availability

Not applicable.

## Abbreviations

WNHSs: World Natural Heritage sites
WNHS: World Natural Heritage Site
WHSs: World Heritage sites
WHKSs: World Heritage karst sites
OUV: Outstanding universal value
UNESCO: United Nations Educational, Scientific and Cultural Organization
WHC: World Heritage Committee
CNKI: China national knowledge infrastructure
WOS: Web of science
IUCN: International Union for Conservation of Nature
GBR: Great barrier reef
GIS: Geographic information system
RS: Remote sensing
